# Cystoid macular edema as a complication of central retinal artery occlusion^☆^

**DOI:** 10.1016/j.ajoc.2024.101998

**Published:** 2024-01-20

**Authors:** Rania Estawro, Neda Abraham, Yousef Fouad, Elodie Bousquet, David Sarraf

**Affiliations:** a Retinal Disorders and Ophthalmic Genetics Division, Stein Eye Institute, University of California Los Angeles, Los Angeles, CA, United States; b Retina Department, Al-Watany Eye Hospital, Cairo, Egypt; c Department of Ophthalmology, Ain Shams University Hospitals, Cairo, Egypt; d Department of Ophthalmology, Lariboisière Hospital, Assistance Publique-Hôpitaux de Paris, University of Paris Cité, Paris, France; e Greater Los Angeles VA Healthcare Center, Los Angeles, CA, United States

**Keywords:** Central retinal artery occlusion, Cystoid macular edema, Optical coherence tomography, En face optical coherence tomography, Silent macular edema

## Abstract

**Purpose:**

To describe the development of cystoid macular edema (CME) as a complication of central retinal artery occlusion (CRAO) in 2 cases.

**Observations:**

The first patient was a 51-year-old female who presented with acute loss of vision in the left eye. Multimodal retinal imaging revealed a CRAO with a perfused cilioretinal artery. CME acutely developed one week after presentation. Cystoid spaces predominantly involved the outer nuclear layer (ONL) on optical coherence tomography (OCT) and completely resolved in two weeks. The second case was a 50-year-old man who presented with acute vision loss in the right eye for 3 weeks. Multimodal retinal imaging illustrated an acute CRAO of the right eye. Four weeks later, visual acuity spontaneously improved to 20/20 and was maintained at 20/20 for more than 2 years. After 28 months, the patient returned with a recurrent drop of vision in the right eye. Cross sectional and en face OCT revealed CME in the right eye without leakage on FA. Cystoid spaces predominantly involved the inner nuclear layer (INL) and resolved with intravitreal anti-VEGF injection combined with carbonic anhydrase inhibitor (CAI) and steroid topical drop therapy.

**Conclusions and Importance:**

CME can rarely complicate both the acute and chronic phase of CRAO. In the acute phase, cystoid spaces were transient and confined to the ONL on OCT. While in the chronic phase, cystoid spaces were confined to the INL on OCT and angiographically silent on FA. Further studies are needed to identify the incidence, underlying pathophysiology and visual prognosis of CME in cases of CRAO.

## Abbreviations

BCVA: Best Corrected Visual Acuity
BRAO: Branch Retinal Artery Occlusion
CAI: Carbonic Anhydrase Inhibitor
CME: Cystoid Macular Edema
CRA: Central Retinal Artery
CRAO: Central Retinal Artery Occlusion
FA: Fluorescein Angiography
INL: Inner Nuclear Layer
IRF: Intra Retinal Fluid
OCT: Optical Coherence Tomography
OD: Right Eye
OS: Left Eye
OU: Both Eyes
PAMM: Paracentral Acute Middle Maculopathy
RAO: Retinal Artery Occlusion
RPE: Retinal Pigment Epithelium
VEGF: Vascular Endothelial Growth Factor

## 1 Introduction

Cystoid macular edema (CME) can be the result of various etiologies including exudative, degenerative and tractional mechanisms.^1^, ^2^, ^3^, ^4^ The location of the intraretinal fluid (IRF) on optical coherence tomography (OCT) can provide a clue to the causative pathway.^5^^,^^6^ Exudative CME due to retinal vascular and inflammatory disorders occurs both in the middle (inner nuclear layer or INL) and outer (outer nuclear layer or ONL) layers.^4^ Degenerative IRF localizes exclusively in the middle retinal layer while tractional IRF (e.g. epiretinal membrane) is identified exclusively in the outer retinal layer.^7^^,^^8^

CME is a common exudative complication of central retinal vein occlusion causing vision loss.^9^ There is commensurate leakage with fluorescein angiography (FA). Anti-vascular endothelial growth factor (VEGF) injection therapy is often necessary to resolve the IRF and improve visual acuity.^10^ However, the detection of IRF as a complication of retinal artery occlusion is very rare.^11^^,^^12^

This case report will describe the multimodal retinal imaging features of 2 cases of CRAO that were associated with the development of IRF. CME complicated the acute phase of CRAO in the first patient and the chronic phase of CRAO in the second patient.

### 1.1 Case 1

A 51-year-old female patient presented to the emergency room with acute vision loss in the left eye upon waking up. Past medical history was significant for hypertension, coronary artery disease, and a stroke that occurred 3 years prior with minimal residual neurological deficit. Snellen visual acuity (VA) was 20/20 in the right eye (OD) and counting fingers at 3 feet in the left eye (OS). Intraocular pressure and anterior segment examination were within normal limits.

**Fig. 1:**
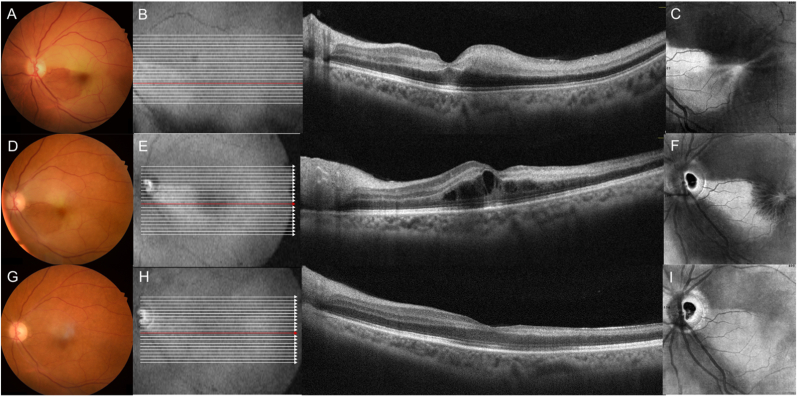
**Multimodal retinal imaging of Case 1 from day 1 (first row), day 7 (second row) and day 21 (third row) of an acute central retinal artery occlusion (CRAO).** Color fundus photography of the left eye on day 1 shows a cherry red spot with ischemic retinal whitening of the macula that spares the region corresponding to the distribution of the cilioretinal artery **(A).** Cross sectional optical coherence tomography (OCT) shows hyperreflectivity in the inner and middle retinal layers involving the temporal macula, and a hyperreflective band involving the middle retinal layer of the nasal parafoveal region consistent with paracentral acute middle maculopathy or PAMM **(B).** En face OCT segmented at the level of the outer retina shows blocked transmission or hyporeflectivity (dark region) corresponding to the area of infarction with relative hyperreflectivity corresponding to the spared nasal region **(C).** On day 7, note the gradual resolution of the ischemia whitening of the macula with color fundus photography **(D)**. Cross sectional OCT shows new onset hyporeflective cystoid spaces within the outer retina **(E),** which assume a petaloid pattern on en-face OCT **(F).** Final follow up of the patient on day 21 shows resolution of the ischemic macular whitening with colored fundus photography **(G)**. Cross sectional and en face OCT shows thinning of the inner and middle retinal layers and resolution of the cystoid fluid **(H** and **I)**. (For interpretation of the references to color in this figure legend, the reader is referred to the Web version of this article.)

Retinal examination and color fundus photography of the right eye were unremarkable. A cherry red spot with ischemic whitening was noted in the macula of the left eye with cilioretinal sparing consistent with an acute CRAO OS (Fig. 1A). Cross sectional OCT showed diffuse hyperreflectivity of the inner/middle retinal layers in the temporal macula consistent with infarction, and a band of hyperreflectivity within the middle retinal layers nasal to the fovea consistent with paracentral acute middle maculopathy (PAMM) (Fig. 1B). En face OCT segmentation of the outer retina showed diffuse hyporeflectivity due to blocked signal transmission with nasal hyperreflectivity due to partial sparing of signal transmission in the area of cilioretinal perfusion (Fig. 1C). A diagnosis of central retinal artery occlusion with a perfused cilioretinal artery was rendered. The patient was sent for urgent cardiovascular consultation and elected to receive intravenous injection of tissue plasminogen activator. Cardiological, neurological, and rheumatological investigations were noncontributory.

At the subsequent follow-up visit 7 days later, Snellen VA improved to 20/120 OS. Cross sectional OCT revealed the development of cystic IRF in the outer retina (Fig. 1E), that assumed a petaloid pattern on en face OCT with outer retinal segmentation consistent with CME (Fig. 1F). A carbonic anhydrase inhibitor (CAI), brinzolamide (twice daily topical eyedrop), was prescribed in an effort to reduce the fluid and improve visual acuity of the left eye.

Two weeks later, VA was further improved to 20/80 OS. Cross sectional OCT showed thinning of the inner and middle retinal layers and complete resolution of the CME (Fig. 1H), which was further confirmed by en face OCT with outer retinal segmentation (Fig. 1I).

### 1.2 Case 2

A 50-year-old man presented with acute vision loss in the right eye for 3 weeks. Past ocular history was remarkable for an 11-year history of branch retinal artery occlusion (BRAO) in the left eye. Medical history was significant for hypertension and hypothyroidism after thyroidectomy to treat thyroid cancer.

**Fig. 2:**
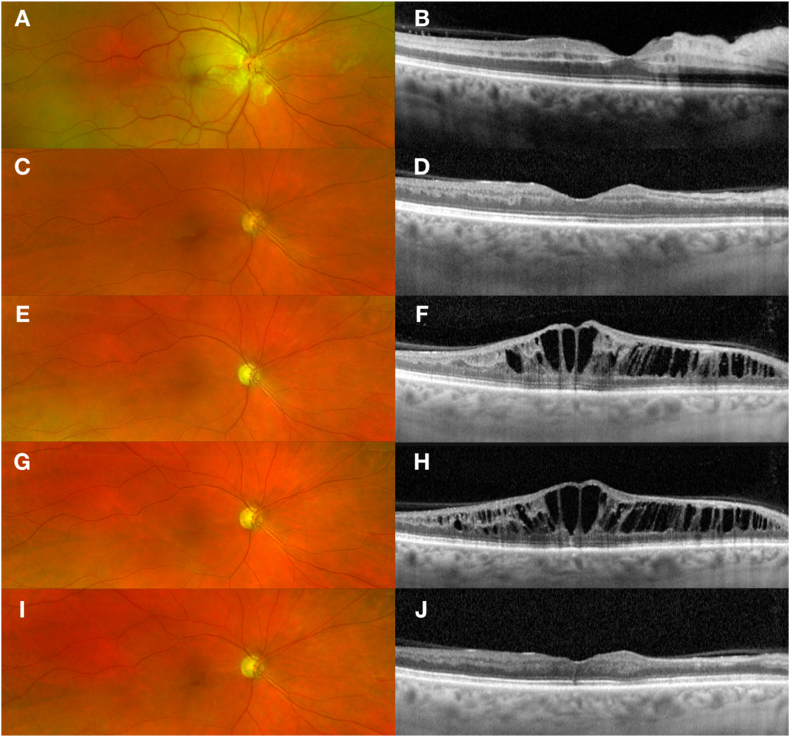
**Multimodal imaging of case 2 at baseline (first row), 4 weeks (second row), 28 months (third row), 29 months (G**–**H), and 33 months (fifth row) after baseline presentation**. At baseline, ultrawide-field color fundus photography of the right eye demonstrates a cherry red spot with ischemic retinal whitening in a peripapillary and macular distribution **(A).** Cross sectional optical coherence tomography (OCT) of the right eye illustrates inner and middle retinal layer hyperreflectivity nasal and middle layer hyperreflectivity temporal corresponding to paracentral acute middle maculopathy (PAMM) **(B).** Four weeks later, color fundus photography OD shows resolution of the ischemic whitening **(C),** and OCT illustrates subsequent thinning of the inner and middle retinal layers **(D).** After 28 months, color fundus photography OD shows optic disc pallor and the development of collateral disc vessels **(E),** and cross sectional OCT OD shows new onset cystoid macular edema confined to the middle retinal layer **(F).** After the first anti-VEGF intravitreal injection (29 months), fundus color photography shows stable collateral vessels on the optic disc in the right eye **(G),** and B scan OCT shows refractory cystoid macular edema **(H).** After the second anti-VEGF intravitreal injection 33 months after the baseline visit, fundus color photography shows stable collateral vessels on the optic disc in the right eye **(I),** and B scan OCT shows complete resolution of degenerative cystoid macular edema (CME) and atrophy of inner retinal layer **(J)**. (For interpretation of the references to color in this figure legend, the reader is referred to the Web version of this article.)

Visual acuity was 20/50 OD and 20/20 OS. Color fundus photography illustrated peripapillary and macular ischemic retinal whitening with a cherry red spot consistent with acute CRAO OD (Fig. 2A). FA was remarkable for delayed arterial filling and prolonged arterio-venous transit time OD. Cross sectional OCT showed hyperreflectivity of the inner and middle retinal layers associated with PAMM lesions OD (Fig. 2B).

The patient was referred for a comprehensive systemic work-up to exclude cardiac, hematological, autoimmune and neurological etiologies, but investigations were only significant for migraine disorder as a potential risk factor. Calcium channel blockers were prescribed by a neurologist to reduce the risk of retinal vasospasm associated with migraine. Four weeks after the acute CRAO, Snellen VA spontaneously improved to 20/20 OD and cross-sectional OCT showed thinning of the inner, and especially, the middle retinal layers OD (Fig. 2D). Visual acuity remained stable for more than 2 years.

**Fig. 3:**
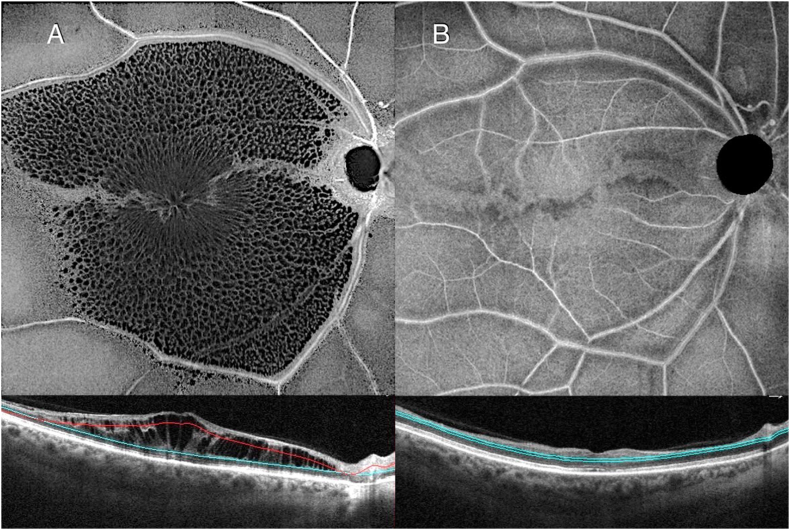
**En face optical coherence tomography (OCT) with the corresponding B-scan of the right eye of Case 2.** At 28 months follow up with new onset cystoid macular edema**,** the middle layer segmentation of the retina highlights the more widespread involvement of cystoid macular edema in a remarkable spoke-like pattern within the foveal region and a reticular pattern in the parafoveal region **(A).** At 33 months follow up, en face and B-scan OCT show complete resolution of cystoid macular edema after the second anti-VEGF intravitreal injection combined with topical CAI and prednisolone acetate eye drops **(B)**.

The patient returned 28 months later complaining of an acute decline of vision in the right eye. Snellen VA was reduced to 20/60 OD and was 20/20 OS. Color fundus photography showed the development of disc pallor and a superior disc collateral OD associated with the old CRAO (Fig. 2E). The macula looked grossly flat and dry on fundus examination, but OCT showed the development of severe cystoid macular edema isolated to the middle retinal layer OD (Fig. 2F). En face OCT segmentation of the INL showed a remarkable retinoschisis-like distribution of IRF, with a central spoke-like pattern in the fovea surrounded with a reticular pattern in the parafoveal region OD (Fig. 3A). FA however failed to demonstrate any evidence of leakage OD and the CME was attributed to a degenerative etiology. A topical CAI drop was prescribed twice daily. However, no anatomical or visual improvement was noted after 4 weeks (Fig. 2H). While an exudative cause of the CME was unlikely given the localization only to the middle retina and the absence of leakage on the FA, an intravitreal anti-VEGF injection was offered given continued vision loss associated with persistent fluid OD. The CME was refractory to the first anti-VEGF intravitreal injection (Fig. 2H). The edema however resolved, with commensurate improvement of VA to 20/20 OD, after the second anti-VEGF intravitreal injection combined with topical CAI twice daily and prednisolone acetate eye drops, 3 times per day (Fig. 2, Fig. 3B) OD.

## 2 Discussion

In this report, we describe the development of cystoid macular edema (CME) in two patients with CRAO. In the first case, CME developed 7 days after acute CRAO, while CME occurred more than 2 years after CRAO in the second case. CME is a very unusual complication of CRAO and has only been rarely reported in this context.^11^^,^^12^ Ng et al. described CME as a complication of acute CRAO.^11^ Of note, CME co-localized to the ONL similar to our first case. Ishizaki et al. retrospectively reviewed 29 patients with retinal artery occlusion and reported the occurrence of CME in 5 cases long after the onset of occlusion in a “schisis like patten” like our second case.^12^ Interestingly, they illustrated 3 cases with accompanying figures. The first case was consistent with a branch retinal artery occlusion (BRAO) with CME involving the ONL in the acute phase, followed by the INL in the chronic phase. The second was a CRAO case that developed CME localized to the INL in the chronic phase. Acute CRAO was also illustrated in Case 3 and CME was isolated to the ONL.^12^

Intraretinal cystoid spaces can be associated with various retinal disorders and may be attributed to various mechanisms, including exudative etiologies due to breakdown of the blood retinal barrier and degenerative pathways due to impairment of Müller cells or the retinal pigment epithelium (RPE).^2^^,^^4^^,^^7^ Müller cells have many critical functions and are an essential element of retinal hydrostasis through facilitation of fluid movement from the intraretinal tissue into retinal capillaries via aquaporin-4 (channel-mediated transport of water) and potassium ions (Kir4.1).^13^^,^^14^

Bringmann et al. proposed that acute retinal ischemia leads to flux of ions into retinal neurons leading to neuronal cell swelling initially, while retinal reperfusion causes down regulation of glial cell conductance with intracellular potassium overload and water movement into Müller cells.^15^ Additionally, Iandiev et al. used immunolabeling to show simultaneous increase of aquaporin-4 and Kir4.1 channel proteins in the outer retina in pathological situations.^16^ Given the anatomical location of the cysts in the outer retina and the transient course of CME, we postulate that Müller cell swelling may explain the transient outer retinal cysts that occur in the context of acute CRAO, as in Case 1 of this report.

OCT can be an important guide to determine the pathway of fluid leakage.^4^^,^^5^^,^^7^ Intraretinal fluid isolated to the middle retina is typically associated with degenerative etiologies (optic atrophy, atrophic age-related macular degeneration, X-linked retinoschisis) while fluid isolated to the outer retina often occurs as a result of tractional causes (e.g., epiretinal membrane or vitreomacular traction).^2^^,^^7^^,^^8^ Exudative CME due to inflammatory disorders, diabetes and retinal vein occlusion tends to be associated with fluid in both the middle and outer retinal layers.^4^^,^^5^^,^^7^ We suspect a degenerative mechanism in Case 2 given the exclusive localization of the IRF to the middle retinal layer (i.e., INL), which was further validated by absence of dye leakage with FA. Additionally, en face OCT segmentation of the INL revealed a characteristic distribution of cystoid spaces in a spoke-like pattern within the foveal region and a reticular pattern in the parafoveal region, which has been recently described in association with X-linked retinoschisis, a degenerative disorder of Müller cells.^17^ The nuclei of Müller cells are located at the level of the INL and perfused by the deep retinal capillary plexus.^13^ Chronic macular ischemia may lead to gliotic alterations of Müller cells and consequent impairment of intraretinal metabolic fluid clearance.

Initially, CME in Case 2 showed resistance to topical CAI treatment and to the first intravitreal anti-VEGF injection. Complete resolution of CME was achieved after the second anti-VEGF injection combined with adjuvant topical CAI and prednisolone acetate eye drops. Asahi et al. proposed a cocktail composed of a steroid, a non-steroidal anti-inflammatory and CAI for treatment of refractory CME.^18^ CAI can be effective in the management of macular edema through enhancement of retinal adhesiveness and increase of the RPE pump function.^19^ Steroids have vasoconstrictive properties that decrease both intracellular and extracellular edema.^1^ Also, steroids have an inhibitory effect on glial cell swelling by stimulating the endogenous adenosine signaling and subsequent opening of extrusion pathways for K+ and Cl-ions.^20^ It is unclear if the resolution of CME in the second case can be attributed to the treatment or the natural course of the disease. Larger longitudinal studies are needed to explore the natural history and proper management of CME in association with chronic CRAO.

## 3 Conclusions

CME is a rare but possible complication of both the acute and chronic phase of CRAO. In the acute stage, cystic spaces were transient and confined to the ONL on OCT and may be the result of acute Müller cell edema. In the chronic stage, CME was confined to the INL with a remarkable spoke-like pattern on en face OCT and may be the result of degenerative pathways associated with Müller cell impairment. Further studies are necessary to identify the incidence and pathophysiology of this complication of CRAO which can adversely affect visual acuity if persistent.

## Financial support

This study was supported by the Research To Prevent Blindness Inc (DS), New York NY.

## Authorship

All authors attest that they meet the current ICMJE criteria for Authorship.

## Declaration of competing interest

The authors declare that they have no known competing financial interests or personal relationships that could have appeared to influence the work reported in this paper.

The authors have no conflict of interest.
